# On the road to becoming a responsible leader: A simulation-based training approach for final year medical students 

**DOI:** 10.3205/zma001111

**Published:** 2017-08-15

**Authors:** Marion Schmidt-Huber, Janine Netzel, Jan Kiesewetter

**Affiliations:** 1 Ludwig-Maximilians-Universität, Munich, former: LMU Center for Leadership and People Management, A47 Consulting Munich, Munich, Germany; 2 Ludwig-Maximilians-Universität, Munich,LMU Center for Leadership and People Management, Munich, Germany; 3 Klinikum der LMU München, Institut für Didaktik und Ausbildungsforschung in der Medizin, München, Germany

**Keywords:** Medical Education, Leadership, Clinical Leadership, simulation-based training

## Abstract

**Background and objective:** There is a need for young physicians to take a responsible role in clinical teams, comparable to a leadership role. However, today’s medical curricula barely consider the development of leadership competencies. Acquisition of leadership skills are currently a by-product of medical education, even though it seems to be a competency relevant for physicians’ success. Therefore, an innovative leadership training program for young physicians was developed and validated. Training conceptualisation were based upon

1. findings of critical incidents interviews (*N*=19) with relevant personnel (e.g. experienced doctors/nurses, residents) and
2. upon evidence-based leadership contents focusing on ethical leadership behaviors.

**Method: **The training consists of four sessions (3-4 hours each) and provided evidence-based lectures of leadership theory and effective leader behaviors, interactive training elements and a simulation-based approach with professional role players focusing on interprofessional collaboration with care staff. Training evaluation was assessed twice after completion of the program (*N*=37). Assessments included items from validated and approved evaluation instruments regarding diverse learning outcomes (satisfaction/reaction, learning, self-efficacy, and application/transfer) and transfer indicators. Furthermore, training success predictors were assessed based on stepwise regression analysis. In addition, long-term trainings effects and behavioral changes were analysed.

**Results:** Various learning outcomes are achieved (self-reported training satisfaction, usefulness of the content and learning effects) and results show substantial transfer effects of the training contents and a strengthened awareness for the leadership role (e.g. self-confidence, ideas dealing with work-related problems in a role as responsible physician). We identified competence of trainer, training of applied tools, awareness of job expectations, and the opportunity to learn from experiences of other participants as predictors of training success. Additionally, we found long-term training effects and participants reported an increase in specific competencies, relevant for effective interprofessional collaboration (active perspective-taking, communication, conflict management, personal competencies).

**Conclusion:** The training of leadership competencies for young physicians seems feasible to develop constructive influence strategies for a successful interprofessional collaboration in early career stages. The simulation-based approach is beneficial for residents to practice leadership behaviour in realistic job situations.

## Zusammenfassung

**Hintergrund und Ziel: **Aufgrund zahlreicher Veränderungen im Gesundheitswesen und der hohen Dynamik im Umfeld klinischer Teams übernehmen Assistenzärzte bereits zu Beginn ihrer medizinischen Laufbahn verantwortungsvolle Rollen in klinischen Teams, die bisweilen auch führungsähnliche Aufgaben beinhalten. Die nationalen wie internationalen medizinischen Ausbildungscurricula tragen dieser Entwicklung derzeit jedoch noch wenig Rechnung und fokussieren vor allem die fachliche Ausbildung des ärztlichen Nachwuchses. Der Erwerb von Führungskompetenzen stellt bislang lediglich eine Art „Nebenprodukt“ der medizinischen Ausbildung dar. Aufgrund dessen berichtet die vorliegende Evaluationsstudie von einem neuartigen Trainingsprogramm für Studierende im Praktischen Jahr (PJ), das die Entwicklung von Führungskompetenzen von Ärzten in den Mittelpunkt rückt. Das evidenzbasierte Trainingskonzept basiert auf

1. Befunden aus Critical-Incident-Interviews (*N*=19) mit klinischem Personal (z.B. erfahrene Ärzte, Assistenzärzte, Pflegepersonal und PJ-Studierende) und
2. bewährten psychologischen Führungsansätzen (v.a. ethikorientierte, authentische und transaktional/transformationale Führung).

**Methode:** Das Trainingsprogramm setzt sich aus vier aufeinander aufbauenden drei- bis vierstündigen Modulen zusammen. Das Konzept beinhaltet Impulsvorträge über bewährte Führungsansätze und effektives Führungsverhalten, interaktive Trainingselemente (z.B. Diskussionen und Reflexionseinheiten) und simulationsbasierte Elemente (Kurzrollenspiele sowie Simulationen mit trainierten Rollenspielern) und fokussiert wirksame Einflussstrategien in der interprofessionellen Zusammenarbeit mit Pflegepersonal. Die Evaluation des Programms fand zu zwei Messzeitpunkten im Anschluss an das Training statt (*N*=37) und erfolgte mittels validierter Items und Evaluationsinstrumente. Ermittelt wurden Facetten des Lerntransfers (Zufriedenheit/Reaktion, Lernen, Selbstwirksamkeit, Transfer/Anwendung) und Transferprädiktoren als Determinanten des Trainingserfolgs.

**Ergebnisse: **Die Ergebnisse weisen auf verschiedene positive Effekte des Trainings hin. Einerseits konnten konkrete Lernerfolge erhoben werden (in Form der selbst berichteten Zufriedenheit, der wahrgenommenen Nützlichkeit der Inhalte und persönlicher Lerneffekte). Weiterhin zeigen sich signifikante Transfereffekte sowie ein verstärktes Bewusstsein für die Anforderungen der Rolle als Führungskraft in klinischen Teams (z.B. in Form von Selbstwirksamkeit, Bewältigungsstrategien im Umgang mit arbeitsbezogenen Herausforderungen in der Rolle des verantwortlichen Arztes). Als Prädiktoren für die Wirksamkeit des Trainings wurden die Kompetenz der Trainer, die Möglichkeit zur Erprobung der diskutierten Inhalte, das Bewusstsein für die beruflichen Anforderungen sowie die Möglichkeit zum Erfahrungsaustausch ermittelt. Darüber hinaus konnten langfristige Trainingseffekte in Form von selbstberichteten Kompetenzentwicklungen in den Bereichen der Perspektivenübernahme, der Kommunikation, des Konfliktmanagements sowie persönlicher Kompetenzen festgestellt werden, die allesamt eine hohe Relevanz zur erfolgreichen Steuerung der interprofessionellen Zusammenarbeit darstellen.

**Schlussfolgerung:** Das Training von Führungskompetenzen für PJ-Studierende hat sich im Rahmen der vorliegenden Studie als praktikabel und wirksam erwiesen, um konstruktive Einflussstrategien für eine erfolgreiche interprofessionelle Zusammenarbeit bereits in der medizinischen Ausbildung zu entwickeln. Der simulationsbasierte Ansatz stellt dabei eine geeignete Methode dar, um Führungsverhalten in einem praxisnahen Setting und anhand von erfolgskritischen Situationen zu trainieren.

## Introduction

As the complexity and dynamics of the healthcare systems grow, leadership competencies of physicians become increasingly important for the success of healthcare teams [1], [2]. There is a need, even for young physicians, to take a kind of leadership role in clinical teams; however, this role usually bears on informal (instead of disciplinary) responsibility [3], [4], e.g. collaborative leadership (cooperation with care staff, resident colleagues, subordinates and other clinical stakeholders) medical responsibility for patients, impact on efficient working climate in their teams, decision making, etc. (for an example see [5], [6]). In daily clinical practice, this role-adoption after completing the exams is expected as a spontaneous individual learning process without systematic guidance. Specific outcome-relevant situations and critical behaviors of medical leaders are not integrated into the medical curriculum thus far [7], [8]. Frugé and colleagues (2010, p. 304) [9] stated that: *“Leadership in medicine […] often is considered a by-product of technical expertise or a combination of technical skill and personal qualities that cannot or should not be a target of formalized educational process.”* In the past the Learning Goal Catalogues of undergraduate medical education little importance gave to integrate leadership competencies [10]. Last year the third version of the CanMEDS Model introduced the leader as a role for future physicians [11] and in the published German National Competency-based Learning Goal Catalogue various learning goals target leadership competencies of future physicians.

Considering the importance of leadership competencies of physicians for successful interprofessional collaboration and patient care [3], [12], it seems important to us to take the growing calls for a more systematic understanding and development of leadership competencies for physicians into account (e.g. [2], [3], [13], [14]). Thus far, studies analyzing specific leadership competencies critical to physicians’ occupational development and success, as well as their structural advancement, are scarce [3], [8]. 

This article deals with a training program that focuses on facilitating the development of selected leadership competencies of medical students in their final year. The overall goal of this program is to strengthen participants’ awareness and selected leadership competencies (particularly perspective-taking, influence strategies, communication, conflict solving) for coping with critical situations as resident physicians during clinical, interprofessional cooperation. In this context, the concept of leadership comprises exerting conscious, goal-oriented social influence on people (subordinates, colleagues and teams) for the purpose of performing shared tasks in pursuit of common goals, and focuses on leading [15]. All discussed situations relied on typical critical incidents young physicians have to cope with during their time as residents, i.e. interprofessional situations or situations with other residents. We explicitly put the focus on differences in role expectations of lateral leadership (exerting influence without formal power) and disciplinary leadership functions as well.

Besides competency development, one aim of the training program is the implementation of an innovative training approach in leadership development. As research demonstrates, behavior is learned more efficiently through training that reflects realistic situations and concrete behaviors into learning experiences [16], [17], [18]. Furthermore, a simulation-based approach is one of the most preferred learning methods of medical students [4], [19]. Therefore, the program provided a high degree of interactivity and simulation-based exercises. In order to assess training effectiveness and the increase of awareness of physicians’ leadership role, we conducted a two-step post-training and long-term-measurement (12-18 months) evaluation design.

The goals of this article are

1. to point out the importance of adding leadership competencies as an integral part of medical education and demonstrate the leadership training program, applied in a German University hospital as a worthwhile expansion of the undergraduate medical education curriculum. Additionally, we
2. present the results of our training evaluation and predictors of training effectiveness, and
3. draw lessons learned with respect to the successful implementation of leadership competency trainings in medical education.

In the following paragraphs, we will provide detailed insights into the development of the training as well as the evaluation design of the program.

## Development of the leadership training program

**Figure 1:**
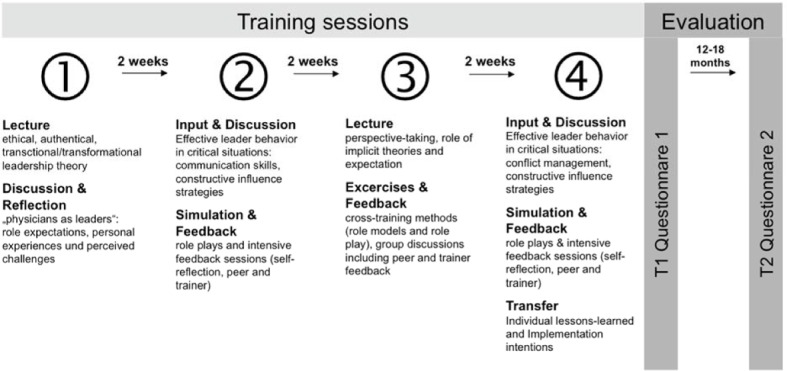
Training program

We conceptualized the training as a multi-part program, consisting of four sessions, for medical students at a German University hospital (e.g. [20]). German medical students are required to work in three different specialties (Internal Medicine, Surgery, and one discipline of their choice) during their final year (the so called “practical year”). The training was an additional offer for all final year students. Students took part per their own initiative and received a confirmation of participation if they completed the entire program. The training program consists of four 3-4 hour training sessions within a six-week period (see figure 1 (Fig. 1)). This training design was chosen to ensure the possibility of additional training in context of daily clinical practice. Methodologically, session one and three contained input parts about effective leadership competencies focusing on ethical and authentic leadership and transactional/transformational leadership theories (e.g. [15], [16]). Beyond lectures, we integrated discussion rounds, and reflection exercises about role expectations and personal experiences in clinical settings. Several interactive tools and exercises (e.g. communication techniques, influencing strategies) were integrated to foster best practice leadership behavior. In sessions two and four the simulation-based approach was utilized with role plays focusing four different critical incidents between physicians and care staff. Every participant acted in the role of a physician and received behavior-based and individual feedback from other participants, the role player and trainers. In order to facilitate learning loops, common lesson-learned-discussions were conducted and implementation intentions were drawn. The training team consisted of two experienced work and organizational psychologists specialized in leadership development, one medical educator, specialized in simulation-based training and two trained actors for the role plays. 

All training contents were based upon evidence-based leadership knowledge and empirical findings. Our key messages in particular, refer to the approach of ethical leadership (e.g. [21]). Ethical leadership emphasizes the responsibility of leaders for human dignity and, at the same time, strives for excellent performance. So, leaders’ drive for success and self-realization is bound by their responsibility for other humans and the environment they live in; *“Act in a way you would like others to act; treat people in a way you would like to be treated; lead in a way you would want to be led”* [22]. 

Beyond evidence-based contents, a training should reflect real performance situations to accentuate positive effects of simulations and role-plays [19]. Therefore, we designed an interview study in order to identify the relevant leadership competencies and assess tailor-made training situations [23]. Following the critical incident technique [24], [25]], structured behavioral event interviews [26] with relevant and interprofessional personnel (*N*=19; experienced doctors/nurses, residents, final-year students) were conducted. All interviews were recorded. Each participant was asked to describe outcome-relevant situations in which experienced or witnessed leadership behaviors of resident physicians were or were not successful (e.g. *“Please think of a situation when a resident physician was in the leading position to solve a critical interprofessional incident. How did he/she act? What was the result of the behavior?”*). Mayring’s content analysis was adopted [27] for data analysis: The authors took transcripts of all answers and put the described behaviors into the categories of an evidence-based model of leadership competencies (see [28]). As a result, we identified the importance of three dimensions of leadership competencies as relevant key aspects of leadership training for final year students:

1. perspective-taking and empathy (*n*=19),
2. communication skills in leading dialogues, e.g. giving constructive feedback, making own positions clear, communicating in a respectful and an appreciative manner (*n*=19) and furthermore
3. conflict resolution and influence strategies in interprofessional teams and across hierarchies (*n*=16).

All interviewees outlined typical clinical situations in which these competencies are relevant to the successful handling of the situation. Our findings are in-line with previous study results which emphasize the importance of communication skills, conflict resolution, empathy, and ethics for the development of resident physicians (e.g. [4], [13], [16], [29]). Therefore, these situations were taken as a basis for the training content and the simulations and role-plays. 

## Method of training evaluation: Evaluation design, questionnaire and data collection

We conducted the training evaluations at two different times after completion of the training program (see figure 1 (Fig. 1)): directly after the fourth session (T1) and 12 till 18 months after the training program (T2). The assessment of training effectiveness is based upon three of the four levels of evaluation by Kirkpatrick, a worldwide standard for evaluating effects of training settings [30], [31]. The framework considers the value of any type of training across four levels:

1. trainees’ *reaction* and response to the program and its content (degree to which participants assess the training favorable, engaging and relevant to their jobs),
2. *learning* effects (degree to which participants acquire the intended knowledge, skills and attitude due to their participation in the training),
3. self-reported *behavior* changes regarding training-related behaviors (degree to which participants apply what they learned during training) and
4. *results* if the training positively impacted the organization.

Latter level was not part of the illustrated evaluation design.

Participants’ reaction to the program and learning effects were mainly measured at T1, whereas self-reported behavior changes were part of measures at T2.

Therefore, T1 questionnaire consisted of the following parts:

1. Four items to evaluate the perceived competence of the trainers (e.g. “The trainers presented the material in a professional manner.”),
2. eleven items taken from the German Learning Transfer System Inventory (GLTSI [32]) to evaluate performance self-efficacy, performance outcome expectations, perceived content validity, and transfer effort performance expectations,
3. ten items taken from the ‘Massnahmen-Erfolgs-Inventar’ (MEI [33]), a German questionnaire to assess the described evaluation criteria of Kirkpatrick (see table 1 (Tab. 1)), and
4. two open-ended questions for comments (positive feedback and negative points).

The participants were asked to rate all items on a 5-point Likert scale of 1 to 5 (1=do not agree, 3=somewhat, 5=definitively agree). To evaluate the effectiveness of the training (based on the criteria of Kirkpatrick), mean scores and standard deviations of the four MEI-scales were computed (see table 1 (Tab. 1)). In order to assess the relevant transfer and success predictors of training effectiveness, we conducted stepwise regression analysis34 for each evaluation criteria measured with the MEI (see table 2 (Tab. 2)).

Data content of the T2 questionnaire referred to transfer and learning effects and real behavioral changes in daily and critical clinical situations (9 items, see table 3 (Tab. 3)). All items comprised a quantitative 5-point Likert scale and open questions referring to concrete behavioral examples (e.g. “After the training, I am more confident in my ability to solve job-related problems in interprofessional collaboration,” [1=do not agree, 3=somewhat, 5=definitively agree]; “Please give an example of a concrete situation…”). We conducted data evaluation with quantitative computation of mean scores, standard deviation, stepwise regression analysis [34] (using SPSS 22.0), and clustering of the answers of all open-ended questions, following the content analysis of Mayring [27]. 

Reliability of scales (Cronbach’s alpha α) proved satisfactory (.73<α< .87), though all scales consist of only 2 items.

## Results

The evaluation questionnaire was administered in 2012 and 2013. We trained three different cohorts with *N*=37 total participants (cohort 1: *N*=10; cohort 2: *N*=17; cohort 3: *N*=10). All participants who completed the training program also completed the evaluation on T1 (37% men, 63% woman), 43% finished our questionnaire at T2 (*N*=16; dropout rate traced back to organizational reasons, e.g. change of the ward or unattainability of participants). 

### Training Evaluation T1

Overall, participants of all three cohorts rated the training as very satisfying, effective and relevant for their jobs as physicians (Kirkpatrick level 1: satisfaction and reaction). Additionally, participants assessed a high degree of learning effects and an improved coping behavior with work demands concerning interprofessional collaboration (Kirkpatrick level 2: learning and self-efficacy). Finally, participants also assessed a high degree of transfer effects (Kirkpatrick level 3: application/transfer). Thus, participants mentioned in the two open questions, that the training supported them to strengthen their empathy, communication skills in leading dialogues and furthermore, conflict solving and influence strategies in critical and high-stake situations of clinical collaboration. Table 1 (Tab. 1) shows all mean scores and standard deviation of the evaluation criteria; all mean scores exceed the score of 4.0 indicating a high benefit of the training program. 

These results are supported by the numerous positive comments (e.g. “motivated, friendly and competent trainers”, “a real high degree of interactivity and possibility to exercise realistic situations”) compared to the marginal comments concerning possible improvements (e.g. “accelerate timeline” or “expand feedback sessions”). The most frequent positive responses applied to the competent trainer team and productive working climate (*n*=35), high degree of interactivity (*n*=29), tailored content and practical hints (*n*=10), and professional organization of the program (n=8). Critical feedback comprised to the length of the program (*n*=4), desire for more simulations of critical collaboration situations, e.g. with the own supervisor or colleagues (*n*=10), and the need for longer feedback sessions (*n*=6).

#### Predictors of training effectiveness

To identify which of the assessed evaluation criteria were substantial predictors for training effectiveness (satisfaction, reaction, learning, self-efficacy, transfer, and effects on behavior), a stepwise regression was analyzed [34] (see table 2 (Tab. 2)). Training satisfaction (*R*²>.63; *p*<.029) and usefulness of training (*R²*>.27, *p*<.012) particularly depend on the behavior of the trainers. However, the awareness for job requirements has an important impact on learning effects, the increase of self-efficacy, and behavioral changes in collaboration (.15<*R²*<.34, *p*<.010). For training transfer, the tools used in training plays a prominent role (*R²*=.27, *p*<.001). Furthermore, the possibility to exchange experiences seems to be an effective intervention method to evoke behavioral advancements (.22<*R²*<.36, *p*<.018).

#### Follow-up evaluation T2

Although mean scores decreased compared to the evaluation scores after the training, the overall result remained satisfactory (see table 3 (Tab. 3)). In sum, at 12 till 18 months post-course, all participants provided positive feedback and confirmed positive training effects for their daily work. Training participants reported amongst others a more professional and target-oriented preparation of conversations and more deescalating and appreciative communication techniques as concrete individual behavior changes. Highest mean scores were assigned for positive effects beyond daily work behavior (*M*=4.00, *SD*=.73), positive changes in collaboration with care staff (*M*=3.94, *SD*=.77), and the usefulness of training content (*M*=3.88, *SD*=.89). Concrete behavioral changes (*n*=32) were reported for active perspective-taking, and improvements in communication style, conflict management, and personal competencies (e.g. self-efficacy and self-reflection skills). 

## Discussion

This study analyzes one of very few trainings to capture long-term training effects of changes in leadership behavior of final year students. The findings are in line with previous studies [10] and show a positive training evaluation at three levels of the evaluation model of Kirkpatrick (satisfaction/reaction, learning and transfer). Therefore, the results strengthen our efforts to implement the development of leadership competencies in medical education as early as possible. 

Results also show that the awareness for the leadership role is – beyond positive assessment results – one of the most important training effects. All participants evaluated the training program, in addition to classical medical education, as effective and important for professional development of future resident physicians.

For the teaching of leadership competencies, it is recommended to use a simulation-based training approach. The methods of simulation and role-play served to strengthen self-efficacy and selected leadership competencies of our participants. This goes in line with findings on the effects of simulation-based training approaches (e.g. [17]) and calls for needs-oriented training designs [8], [23]. Furthermore, the high degree of interactivity facilitated the process of learning from experiences of other participants, and discussions about role expectations in clinical teams. 

Additional evaluation results show that tailor-made exercises, visualizing job requirements concerning leadership situations, and discussion of useful tools and methods are worthwhile and should be implemented as training methods in leadership development programs. We therefor recommend to conduct critical incident interviews prior to the conceptualization of trainings contents. Our results show that satisfaction and learning effects also depend on trainers’ expertise. Training implementation needs to focus on professional leadership trainers who can also act as role-models, give practical hints, and initiate coaching techniques to solve practical challenges (cf. [35]). 

In this article, we also demonstrated the importance of integrating leadership competencies in medical education curricula, as also claimed from other authors [36]. Participants reported substantial effects for the training quality even after 12 till 18 months post-course. Students reported spill-over effects of training experiences in communication style and conflict solving strategies for working behavior beyond leadership situations (e.g. concerning dialogues with medical leaders or colleagues). This shows, that our training program addresses an important learning need in medical education and indicates, that leadership competencies should be integrated in Learning Goal Catalogues of undergraduate medical education [2]. The slightly decline of the training effects after T2 is a typical phenomenon in training evaluation and due to effects of environment, motivation and pre-training self-efficacy [37]. 

## Limitations

The study design includes some shortcomings: Although representative, the number of participants was rather small. In the end, we couldn’t fill all possible training places. One of the biggest challenges to the participants was visiting the training within a full schedule and enabling participation for all 4 training sessions. Furthermore, the participation was voluntary and therefore, a lot of students showed no interest. However, 37 interested, committed, and learning-oriented students participated in all training sessions, which is arguably a good result. Nevertheless, the number of participants should be raised in order to obtain more substantial evidence for the positive effects of the program.

This study used a questionnaire methodology focused on self-assessments of the participants. Therefore, the quality of results depends on participants’ memory and honesty, and can be increased as results of answering biases, e.g. social desirability, recency effects, or memory biases (e.g. see [38]). The cross-sectional evaluation design evokes potential methodical artefacts which can lead – besides the motivated group of participants – to an overestimation of training effects (see [39]). Therefore, we conducted the follow-up measurement to assess transfer effects of the training. However, the influences of these limitations are reduced via the homogeneity of good results throughout the different evaluation levels and cohorts.

Finally, the next implementation should, therefore, add other-ratings of participants’ behaviors in clinical situations (e.g. nursing personnel or colleagues) to minimize self-serving biases and integrate an initial measurement of leadership competencies before participation in the training.

## Conclusion

This article analyzed the effect of a simulation-based training approach in the leadership development of final year students. Setting-up training content on critical incidents interviews facilitated a tailor-made and practice-oriented training program, which was evaluated as successful, relevant, satisfying, and effective. Self-efficacy of participants increased, and concrete behavior changes in clinical situations were reported by most participants. Moreover, the training generated spill-over effects beyond leadership situations for successful and effective interprofessional collaboration. For future training intentions, we recommend a well-considered selection of experienced and leadership-focused trainer team, tailor-made training content, and a high degree of interactivity (e.g. simulation, role-play, group discussions, short coaching, and feedback module). Further training programs, evaluation studies and subsequent funding are needed to assess the efficacy of this training approach for leadership competency development in medicine.

## Funding

This work was supported by the Research Fund “Hildegard Hampp Humanitas” of the University hospital of LMU Munich. The authors won the prize for “Young Lecturers 2014”, awarded from the German Society of Medical Education (“Preis junger Lehrender 2014”, GMA Gesellschaft für Medizinische Ausbildung) and dedicated the prize to the preparation of the manuscript.

## Ethical approval

Ethical approval was obtained by the responsible Ethical Committee of LMU Munich.

## Competing interests

The authors declare that they have no competing interests.

## Figures and Tables

**Table 1:**
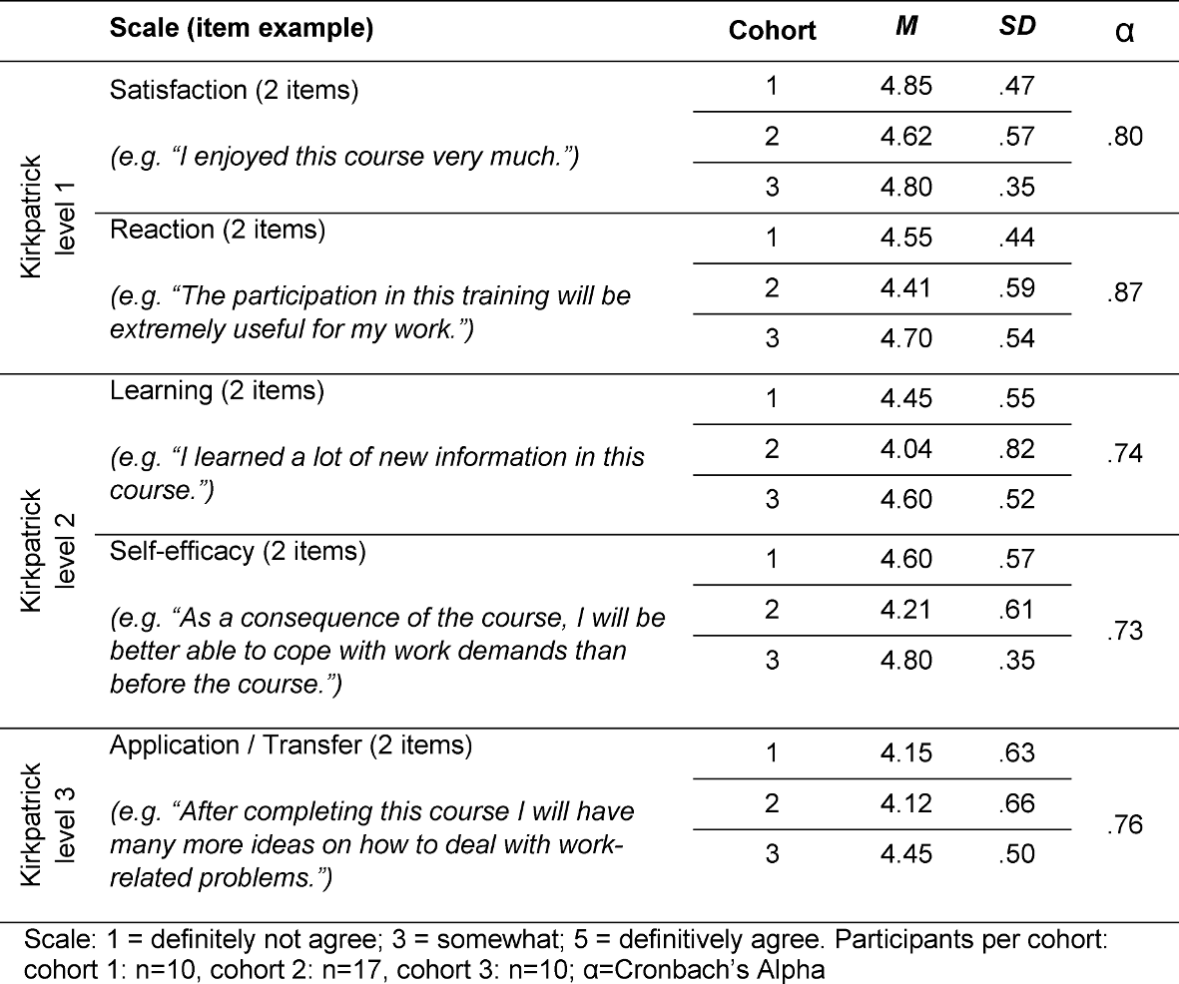
Descriptive statistics of evaluation items T1 (“Maßnahmen-Erfolgs-Inventar” MEI).

**Table 2:**
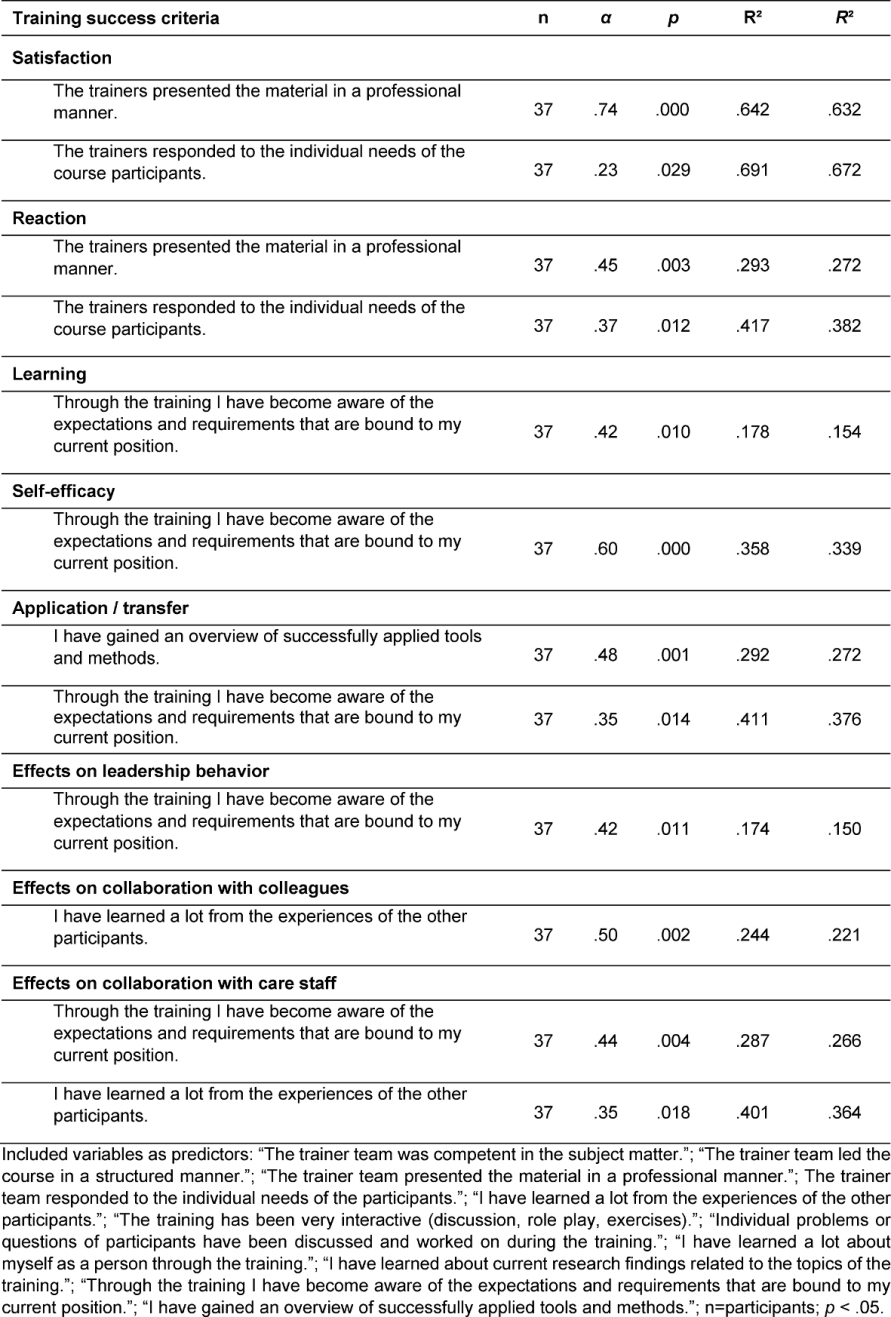
Stepwise regressions of training success criteria based upon German Learning Transfer System Inventory.

**Table 3:**
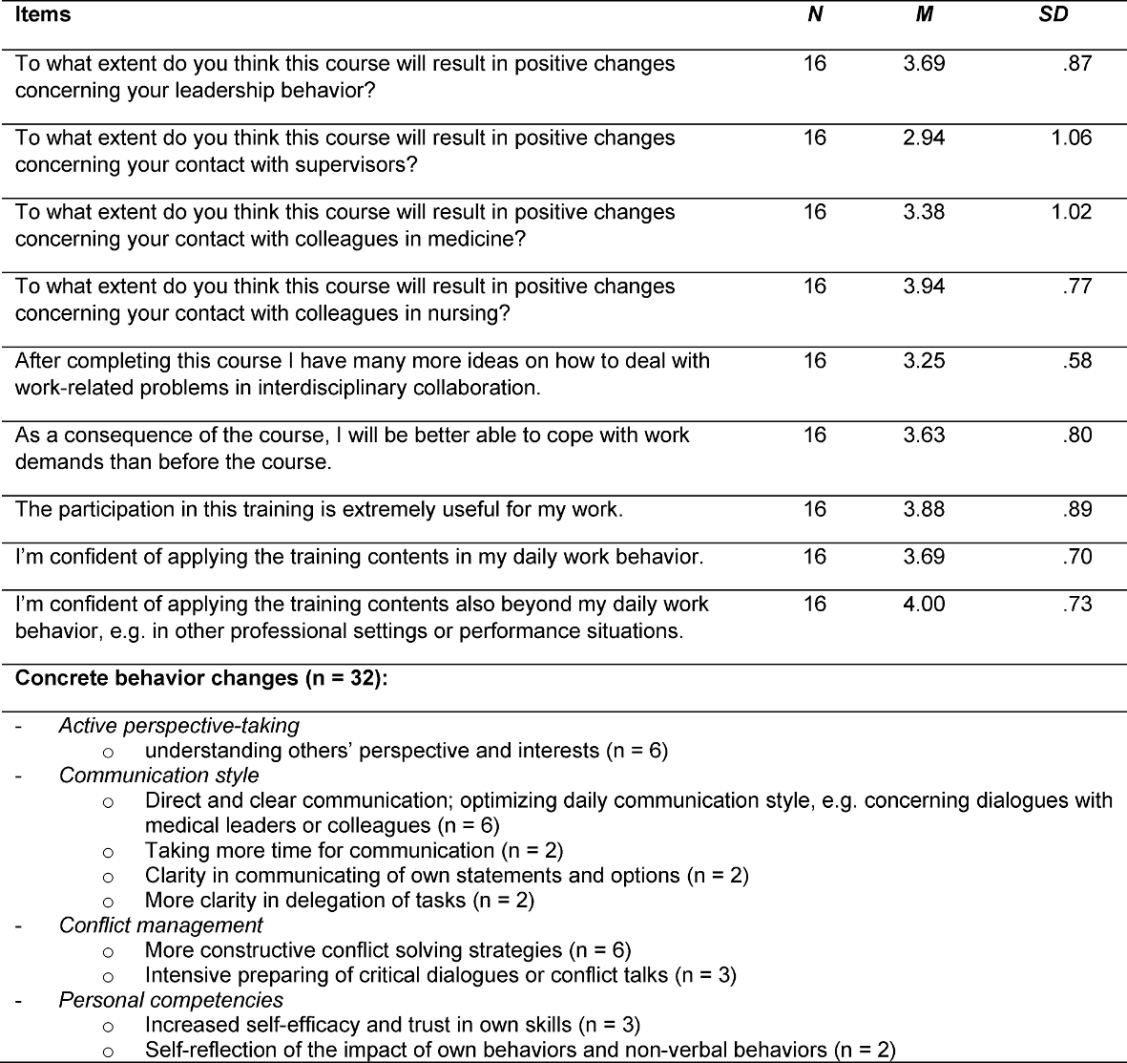
Evaluation results of T2.
